# Focused Ultrasound and RadioTHERapy for non-invasive palliative pain treatment in patients with bone metastasis: a study protocol for the three armed randomized controlled FURTHER trial

**DOI:** 10.1186/s13063-022-06942-1

**Published:** 2022-12-29

**Authors:** Derk J. Slotman, Marcia M. T. J. Bartels, Cyril J. Ferrer, Clemens Bos, Lambertus W. Bartels, Martijn F. Boomsma, Erik C. J. Phernambucq, Ingrid M. Nijholt, Alessio G. Morganti, Giambattista Siepe, Milly Buwenge, Holger Grüll, Grischa Bratke, Sin Yuin Yeo, Roberto Blanco Sequeiros, Heikki Minn, Mira Huhtala, Alessandro Napoli, Francesca De Felice, Carlo Catalano, Alberto Bazzocchi, Chiara Gasperini, Laura Campanacci, Julia Simões Corrêa Galendi, Dirk Müller, Manon N. G. J. A. Braat, Chrit Moonen, Helena M. Verkooijen, on behalf of the FURTHER consortium

**Affiliations:** 1 Division of Imaging and Oncology, University Medical Centre Utrecht, Utrecht, The Netherlands; 2 Department of Radiology, Isala Hospital, Zwolle, The Netherlands; 3 Department of Radiation Oncology, Isala Hospital, Zwolle, The Netherlands; 4 DIMES, Alma Mater Studiorum - Bologna University, Bologna, Italy; 5 Radiation Oncology, IRCCS Azienda Ospedaliero-Universitaria Di Bologna, Bologna, Italy; 6 Institute of Diagnostic and Interventional Radiology, Faculty of Medicine and University Hospital Cologne, University of Cologne, Cologne, Germany; 7 Department of Radiology, Turku University Hospital, Turku, Finland; 8 Department of Oncology, University of Turku and Turku University Hospital, Turku, Finland; 9 Department of Radiological, Oncological and Pathological Sciences, Sapienza University of Rome, Rome, Italy; 10 Diagnostic and Interventional Radiology, IRCCS Istituto Ortopedico Rizzoli, Bologna, Italy; 11 3Rd Orthopaedic and Traumatologic Clinic Prevalently Oncologic, IRCCS Istituto Ortopedico Rizzoli, Bologna, Italy; 12 Institute of Health Economics and Clinical Epidemiology, Faculty of Medicine and University Hospital of Cologne, University of Cologne, Cologne, Germany

**Keywords:** (MeSH): Cancer pain, Palliative care, Palliative therapy, Pain management, Neoplasm metastasis, Bone metastases, Radiotherapy, Radiation oncology, High-intensity focused ultrasound ablation, Magnetic resonance imaging interventional

## Abstract

**Background:**

Cancer-induced bone pain (CIBP), caused by bone metastases, is a common complication of cancer and strongly impairs quality of life (QoL). External beam radiotherapy (EBRT) is the current standard of care for treatment of CIBP. However, approximately 45% of patients have no adequate pain response after EBRT. Magnetic resonance image-guided high-intensity focused ultrasound (MR-HIFU) may improve pain palliation in this patient population. The main objective of this trial was to compare MR-HIFU, EBRT, and MR-HIFU + EBRT for the palliative treatment of bone metastases.

**Methods/design:**

The FURTHER trial is an international multicenter, three-armed randomized controlled trial. A total of 216 patients with painful bone metastases will be randomized in a 1:1:1 ratio to receive EBRT only, MR-HIFU only, or combined treatment with EBRT followed by MR-HIFU. During a follow-up period of 6 months, patients will be contacted at eight time points to retrieve information about their level of pain, QoL, and the occurrence of (serious) adverse events. The primary outcome of the trial is pain response at 14 days after start of treatment. Secondary outcomes include pain response at 14 days after trial enrolment, pain scores (daily until the 21st day and at 4, 6, 12 and 24 weeks), toxicity, adverse events, QoL, and survival. Cost-effectiveness and cost-utility analysis will be conducted.

**Discussion:**

The FURTHER trial aims to evaluate the effectiveness and cost-effectiveness of MR-HIFU—alone or in combination with EBRT—compared to EBRT to relieve CIBP. The trial will be performed in six hospitals in four European countries, all of which are partners in the FURTHER consortium.

**Trial registration:**

The FURTHER trial is registered under the Netherlands Trials Register number NL71303.041.19 and ClinicalTrials.gov registration number NCT04307914. Date of trial registration is 13–01-2020.

**Supplementary Information:**

The online version contains supplementary material available at 10.1186/s13063-022-06942-1.

## Background

Cancer-induced bone pain (CIBP) due to bone metastases often results in substantial deterioration of quality of life (QoL) in patients with advanced cancer [1, 2]. The mainstay of treatment of CIBP is oral analgesics (mostly opioids), in some cases stabilization or fixation surgery and in most cases external beam radiotherapy (EBRT) [3–5]. Although EBRT is a well-established treatment option, not all patients experience pain relief after this treatment. A recent systematic review has shown that approximately 45% of patients do not respond to EBRT and if patients do respond, it can take up to 6 weeks before adequate pain relief is reached. [6–8] Moreover, approximately 50% of initial responders experience recurrent pain, for which re-irradiation is only effective in 58% of patients [9, 10]. Fast and effective pain control is paramount to optimize QoL in palliative cancer care. Magnetic resonance image-guided high-intensity focused ultrasound (MR-HIFU), as an alternative or addition to EBRT, may improve pain palliation treatment in this patient population, by increasing the percentage of responders, and decreasing the time to response [11, 12].

MR-HIFU is a non-invasive treatment modality which can deliver acoustic energy to heat target tissue to ablative temperatures [11, 13]. One of many potential applications of MR-HIFU is in palliative pain treatment for CIBP, in which the pain palliation mechanism is hypothesized to be through ablation of periosteal nerves and tumor debulking. Previous studies have shown that pain response may occur within 3 days after MR-HIFU treatment, and response rates are promising with pain responses ranging from 67 to 88% of patients [14–21]. Moreover, combining MR-HIFU with EBRT may have a complementary or even synergistic effect on pain response [22–25]. In an earlier study, the feasibility of combined treatment was proven. [25]

Still, high-quality evidence and context are needed to determine the role of MR-HIFU in the first-line treatment options of patients with CIBP. The FURTHER consortium sets out to provide this evidence and to evaluate the effectiveness and cost-effectiveness of MR-HIFU (alone or in combination with EBRT) as a palliative treatment option for patients with CIBP caused by bone metastases. To date, no randomized controlled trials have been performed to compare the current standard of care (EBRT) to MR-HIFU or combined treatment. Therefore, we designed a three-armed randomized controlled trial with the objective to compare Focused Ultrasound and RadioTHERapy for Noninvasive Palliative Pain Treatment in Patients with Bone Metastases—The FURTHER trial.

## Methods

### Study design

The FURTHER trial is a multicenter three-armed randomized controlled superiority trial, performed in six hospitals in four European countries, all of which are partners in the FURTHER consortium. The trial will be coordinated from the UMC Utrecht, with a steering group consisting of representatives of all institutions and an external advisory board. The design and report of this protocol follow the Standard Protocol Items: Recommendations for Interventional Trials statement. [26]

**Fig. 1:**
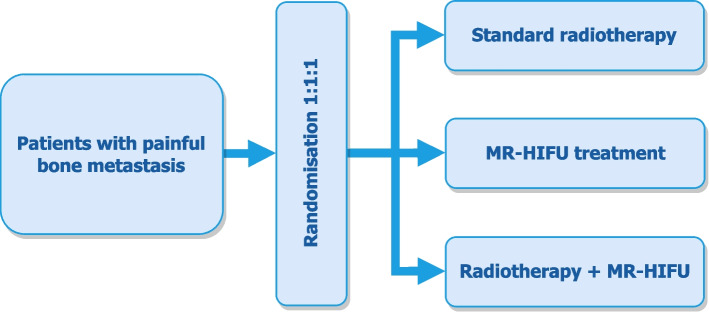
Flowchart of the FURTHER-trial randomization design

After inclusion, patients are randomized in a 1:1:1 ratio to one of the three intervention arms and will receive either standard EBRT, MR-HIFU only, or combined EBRT and MR-HIFU (Fig. 1). Treatment will be delivered as soon as possible. A total of 216 patients will be randomized, 72 patients into each arm of the trial.

### Intervention allocation

Randomization will be done centrally with a computer-generated sequence in Castor EDC [27]. Variable block randomization will be applied as allocation concealment mechanism. Randomization is stratified by institution and planned EBRT fractionation schedule. Patients and doctors will not be blinded to treatment and treatment allocation will not be concealed. Concealment of treatment allocation is not possible due to the evident differences between both MR-HIFU and EBRT interventions. Randomization will be performed during or after physical consultation on the Radiotherapy Department. Shortly after randomization, the patients will be informed about the randomization outcome and randomization arm-specific procedures, and treatment preparations will be started.

### Objectives

The aim of the FURTHER trial is to evaluate the effectiveness and cost-effectiveness of MR-HIFU (alone or in combination with EBRT) compared to EBRT alone for palliation of CIBP. The primary endpoint of this trial is patient-reported pain response 14 days after the start of treatment (Table 1). As MR-HIFU has been shown to be particularly fast acting (within 3 to 7 days [14–21]), compared to EBRT (4–6 weeks [6–8]). It is expected that patients suffering from cancer-induced bone pain could mainly benefit from MR-HIFU in the early weeks after treatment. Therefore, the primary endpoint was set at 14 days.

**Table 1:** Primary and Secondary outcomes of the FURTHER-trial, a trial looking at the impact of treatment with MR-HIFU with or without radiotherapy on patients with painful bone metastases

**Primary outcomes**	
1.1. Pain response 14 days after completion of treatment	Assessed using the BPI and patient pain diary. Used to assess the short-term effectiveness of treatment
1.2. Pain response at 14 days after inclusion	Assessed using the BPI and patient pain diary. Used to assess the effectiveness of treatment taking hospital logistics and planning into account
**Secondary outcomes**	
2.1. Patient-reported pain scores	Assessed using the BPI and patient pain diary during the first 21 days, at 4 and 6 weeks and 3 and 6 months following treatment
2.2. Physician reported toxicity	Assessed by telephone call at 3 days; 1, 4, and 6 weeks; and 3 and 6 months following completion of treatment according to the Common Terminology Criteria for Adverse Events (CTCAE) version 5.0
2.3. Patient-reported quality of life	Assessed using the EORTC QLQ BM22, C15-PAL, EQ-5D-5L, and PGIC at baseline; 1, 2, and 4 weeks; and 3 and 6 months following completion of treatment
2.4. Local tumor control	Assessed using CT and/or MRI imaging at patient discretion at 3 and/or 6 months after completion of treatment
2.5. Levels of anxiety and depression	Assessed among patients, patients’ partners, and caregivers by the Hospital Anxiety and Depression Scale (HADS) and EDIZ-list at baseline; 1, 2, 4, and 6 weeks; and 3 and 6 months following completion of treatment
2.6. Cost-effectiveness of the treatment	Assessed as cost per responder and cost per quality-adjusted life years (QALY), from a payer perspective, at 6 months of follow-up

Pain response will be based on the numeric rating scale (NRS) and the pain severity index calculated from the Brief Pain Inventory (BPI) questionnaire [28, 29]. In addition, analgesic and anti-neuropathic drug use is recorded, and all opioid analgesics are expressed as the oral morphine equivalence dose (OMED). The primary endpoint of the trial will follow the International Consensus on Palliative Radiotherapy Endpoints for Future Clinical Trials in Bone metastases [30]. In accordance with the consensus, pain will be assessed by the worst pain score over the previous 3 days. Patients will be categorized as responders when either a reduction of pain score of at least 2 points without increase of analgesic intake is achieved, or a reduction of analgesic intake of at least 25% is accomplished without an increase of pain score at the treated site. All other patients will be categorized as non-responders.

As adequate palliation should also include sufficient long-lasting pain relief, long-term outcomes are added as secondary endpoints (Table 1). They are set as primary endpoints as long-term outcomes are already managed relatively well in current clinical practice by EBRT. One of the several possible outcomes of this trial is that MR-HIFU-only does allow short-term pain palliation, but fails in the long term. For this situation, a combination arm of MR-HIFU + EBRT is added to the trial as an alternative treatment strategy that potentially combines the capabilities of both individual treatments, at the expense of cost-effectiveness. In this manner, we hope that this trial will help clarify whether the potential benefits of MR-HIFU have added value to the current standard of care, by applying it either as substitute or addition to EBRT in some patients. Secondary endpoints (Table 1) include QoL at 1, 2, and 4 weeks and 3 and 6 months after the start of treatment using validated questionnaires (EORTC QLQ BM22, EORTC C15-PAL, EQ-5D-5L, and PGIC) [31–35]. Furthermore, patient-reported pain response at 14 days after inclusion is added as secondary endpoint, as this will reflect the differences in complexities in planning EBRT versus MR-HIFU. We will also compare the evolution of pain scores between MR-HIFU and EBRT treatment groups (time to pain palliation, time to pain progression, duration of pain palliation). Cost-effectiveness and cost-utility analyses will be performed. Although the primary goal of both interventions will be pain palliation, we will also evaluate local control. Local control will be assessed at 3 and/or 6 months for patients for whom MR of computed tomography (CT) imaging is available. The complete list of primary and secondary endpoints is shown in Table 1.

### Patient selection and follow-up

The study population will consist of male and female adults (age ≥ 18 years) with non-vertebral painful bone metastases that are accessible for MR-HIFU and EBRT who are able to give a written informed consent. Patients will be enrolled at the departments of Radiation Oncology of all participating centers. The radiation oncologist will approach potentially eligible patients who meet the in- and exclusion criteria for study participation (Table 2). (S)he will shortly explain the study (design) and the three treatment strategies (EBRT only, MR-HIFU only, or combined EBRT and MR-HIFU). When patients express interest, they will receive more detailed information about the study. The investigator or an authorized delegate will check with patients whether they have understood the aim and content of the study. Then, patients will be requested to sign a written informed consent.

**Table 2:** Inclusion and exclusion criteria of the FURTHER trial

Inclusion criteria	Exclusion criteria
Age ≥ 18 years	Previous surgery on the target location
Patient capable of giving informed consent	Neurological symptoms due to nerve involvement of target lesion
Referral to radiotherapy department due to painful metastatic bone lesion (NRS ≥ 4)	Need for surgery of targeted location due to (impending) pathological fracture
Pain from the target lesion is distinguishable from other lesions *	Unavoidable critical structures or dense tissues in the target area ***
Target lesion location is accessible for MR-HIFU and EBRT **	Curative intention of treatment plan
Target lesion is visible on pre-treatment MR or CT imaging, with a maximum diameter of 8 cm	Contraindications MRI or sedation
Participant able to fit in the MRI gantry	Participant enrolled in another clinical interventional study related to bone metastases treatment or pain relief treatment
Reasonable performance score (KPS ≥ 50% or Zubrod/ECOG/WHO < 3)	Clinically relevant medical history or physical findings that could interfere with the patient's safety as judged by the treating physician
Life expectancy ≥ 3 months	

^*^Solitary painful metastatic bone lesion or multiple metastatic lesions with one predominantly painful target lesion (≥ 2 points higher pain score than other lesions)

^**^For example, extremities, pelvis (os pubis, os ilium, os ischium, sacrum, acetabulum), shoulders, in selected cases ribs and sternum (if no lung tissue in HIFU beam path)

^***^As judged by the operator, e.g., nerve bundles, skin, extensive scarring, non-targeted bones, air (e.g., hollow viscera), and (external) fixation device

*ECOG* Eastern Cooperative Oncology Group, *KPS* Karnofsky Performance Score, *HIFU* high-intensity focused ultrasound, *EBRT* external beam radiotherapy

Given the urgent need for rapid pain relief, patients will be allowed to make a decision for participation in the study and sign an informed consent immediately after receiving the information. The researcher will randomize the patient using the online Castor EDC database [27]. The treatment arm allocation will immediately be shown. Patients will be treated according to the treatment arm they are assigned to.

**Fig. 2:**
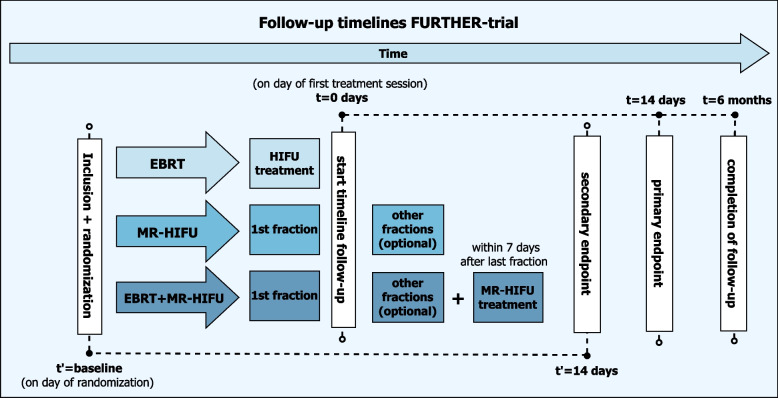
Schematic overview of the follow-up timelines for the FURTHER trial illustrating the start and end of follow-up. In addition, the primary endpoint and secondary endpoint 14 days after inclusion are visualized. For all three arms, i.e., external beam radiotherapy (EBRT), magnetic resonance-guided high-intensity focused ultrasound (MR-HIFU), and EBRT + MR-HIFU, the follow-up is started after the first treatment session

To retrieve information about their level of pain, QoL, and (serious) adverse events, patient outcome measures will be assessed at several time points during a follow-up period of 6 months (Table 3). Starting on the treatment day, a paper-based patient diary will be used for self-reporting of pain levels and pain medication use during the first 21 days following the start of treatment, and at 1, 3, and 6 months. The paper diary and questionnaires will be supplied and explained to patients prior to the treatment. Furthermore, patients will also be interviewed or contacted by telephone at baseline, and at 1, 2, 4, and 6 weeks and 3 and 6 months to assess pain response, patient-reported QoL, and (serious) adverse events. A schematic overview of the follow-up timelines for each randomization arm is illustrated in Fig. 2. The paper-based diary and questionnaires are collected by the local partner institutions and entered into the web-based central Castor EDC database. A data management plan is in place to ensure data quality and a dedicated data-management team located at the UMC Utrecht will ensure data security, coding, and storage. Quality and completeness of data will be monitored by an independent team of trained data monitors.

**Table 3:** Follow-up timeline in the FURTHER trial

	Ti	T0	T1	T2	T3	T4	T5	T6	T7	T8
	Incl	Tx	3d	7d	14di	14d	4w	6w	3 m	6 m
Informed consent	**x**									
Baseline data	**x**									
Patient diary *		**x**	**x**	**x**	**x**	**x**				
Brief Pain Inventory	**x**						**x**	**x**	**x**	**x**
Assessment of adverse events (by telephone)			**x**	**x**	**x**	**x**	**x**	**x**	**x**	**x**
Quality of life questionnaires **		**x**		**x**		**x**	**x**	**x**	**x**	**x**
Anxiety and depression in patient/partner/caregiver (HADS + EDIZ)		**x**		**x**		**x**	**x**	**x**	**x**	**x**
Patient Global Impression of Change score				**x**	**x**	**x**	**x**	**x**	**x**	**x**
CT and/or MRI ***		**x**							**x**	**x**

^*^First 21 days after treatment. Contains daily BPIs and weekly PROMs

^**^EORTC QLQ BM22, EORTC QLQ C15-PAL, EQ-5D-5L

^***^At the patient discretion

Patients are free to leave the study at any time. Patients will be followed until death or end of study (6 months). To reduce the impact of patient death on loss to follow-up, the inclusion criteria “life expectancy > 3 m” was added. To further minimize missing data, patients will be actively contacted by phone on scheduled times to remind them of filling in the questionnaires. No specific dropout criteria are defined. The investigator can decide to withdraw a patient from the study for urgent medical reasons. Patients will be considered non-responders when they will be referred to alternative palliative treatments of the treated metastasis (such as (re)irradiation, radiopharmaceuticals, surgery, cryotherapy, radio-frequency ablation or nerve blocks, or MR-HIFU in the EBRT arm).

Since this study concerns a one-off treatment, patients will return to their general practitioner or referring oncologist. All participating centers have insurance that covers damage to research subjects through injury or death caused by the study. The insurance applies to damage that becomes apparent during the study or within 4 years after the end of the study.

### Study procedures

#### Radiotherapy treatment

Control patients will undergo standard radiotherapy for painful bone metastases. The radiation schedule is at the discretion of the treating radiation oncologist. The radiation oncologist may decide to administer a single fraction EBRT of 8 Gy, a multi-fraction regime of 20 Gy in 5 fractions, 24 Gy in 6 fractions, or 30 Gy in 10 fractions. Fractions are standardly delivered once daily 5 days a week, making the total treatment length variable and span multiple days for multi-fraction treatment plans. A planning CT with or without contrast agent in treatment position will be taken for target delineation. Treatment plans may be delivered using intensity-modulated radiation therapy (IMRT), volumetric modulated arc radiotherapy (VMAT), or stereotactic body radiation therapy (SBRT) technique. Plans will be accepted if at least 90% of the planning target volume (PTV) will receive 95% of the prescribed dose (V90 > 95%). Typically, a 10-mm isotropic PTV margin will be used. A maximal 3D dose of 110% will be allowed. The maximum allowed dose in organs at risk will be determined according to the local institution’s protocol. Position verification is at the discretion of the treating radiation oncologist in consultation with the clinical physicist. It is encouraged to use online position verification by use of cone beam CT at every fraction.

#### MR-HIFU treatment

MR-HIFU treatment will be delivered on a clinical MR-HIFU system (Sonalleve™ System V2, Profound Medical Corp, Mississauga, Canada, or Exablate™ 2100, Insightec, Tirat Carmel, Israel), integrated into a 1.5- or 3-T MR scanner (Achieva™, Ingenia™, Philips Healthcare, Best, The Netherlands, or Signa™, GE Healthcare, Milwaukee, WI, USA). MR-HIFU treatment will aim at ablating the periosteum followed by ablation of the bone metastasis if feasible and safe. HIFU treatment will be performed in accordance with the international consensus paper. [12]

During treatment preparation, the skin overlying the site of interest will be shaved and premedication (consisting of analgesics) will be administered. The procedure will be done using either procedural sedation and analgesia or general anesthesia. Patients will be positioned to obtain an HIFU beam path as perpendicular to the cortical bone surface as possible. After treatment planning and patient positioning, a low-energy sonication test will be performed to confirm reachability and correct for potential HIFU beam path distortion. Maintaining a temperature of at least 55 °C for 1 s as measured by MR thermometry in front of the cortical bone is considered to deliver a thermal dose high enough to achieve adequate ablation of the periosteum [36, 37]. During treatment, real-time temperature mapping will be used to monitor whether this goal was achieved and to determine the completion of treatment. Preferably full lesion surface coverage will be achieved by systematically sonicating treatment cells in a contiguous way, respecting system-recommended cooling times between sonications. After the HIFU procedure, a contrast-enhanced T1w-scan using a gadolinium-based standard MR contrast agent will be acquired to evaluate the treatment effect. The patients are then transferred to the recovery room or patient ward for medical supervision. When the patient has recovered from procedural sedation and no complications have occurred, the patient can be discharged on the same day. Alternatively, patients may, at the discretion of the treating physician, stay overnight at the hospital. The MR-HIFU treatment exists in one session, and the treatment duration is several hours, depending on lesion size and location. The MR-HIFU procedure has a good safety profile: there is a small risk of minor treatment-related side effects including pain in the treated area, first-/second-degree skin burns, and bruising. Severe side effects are extremely rare and include necrosis of nontarget tissue, ulcerating skin burns, and anesthetics-related complications [13]. These side effects will be monitored during hospitalization and follow-up phone calls. If MR-HIFU treatment does not alleviate pain within 4 weeks after treatment, patients will be offered standard care (EBRT).

#### Combined treatment

Patients allocated to the combined intervention arm will undergo both interventions as described above. In our previous research, it was shown that the combined treatment of EBRT and MR-HIFU is feasible and safe [25]. Clinical outcomes were promising and need to be further assessed in this comparative trial.

In the combination treatment arm, both treatments will be planned as soon as possible following inclusion (preferably within one week), to ensure rapid pain palliation. The pre-treatment appointments will be planned in a way that is least burdensome for the patient. Therefore, flexible planning of preoperative screening and MR-HIFU treatment to minimize extra hospital visits is encouraged. To ensure an efficient workflow for the combined arm, a weekly available MR-HIFU slot is advised. The total treatment length is defined as the time between the first and last treatment session and depends on EBRT fractionation schedule and MR-HIFU availability.

#### Analgesic medication

Patients may receive pain medication and/or dexamethasone as required by their symptoms, both before and after the EBRT and/or MR-HIFU treatment. The amount of pain medication used is part of the primary and secondary endpoints of the study and will be recorded at baseline before treatment and during follow-up. During the MR-HIFU treatment, the patient will receive hypnotic and analgesic agents under the direct supervision of an anesthesiologist or sedationist for pain control and to ensure a stable patient position during treatment. Drug selection will be based on practitioner preference.

### Sample size calculation

The study is powered to detect a difference in proportions of patients with pain response of the treated bone metastasis at 14 days after the start of treatment. For this purpose, the two MR-HIFU arms will be combined, and the sample size will be determined on a 2:1 comparison. We calculated we need 72 patients in each arm to achieve a power of 90%, using a one-sided alpha of 5% and assuming a 10% post-randomization dropout using R version 4.0.4 (R foundation for statistical computing; https://R-project.org/). We assume that the response rates during this trial will be similar to previous studies. Therefore, we base this calculation on previous research showing that the proportion of patients with successful pain palliation at 14 days following the start of treatment is 0.69 for MR-HIFU and 0.40 for EBRT [9, 14]. We conservatively assume that 10% of patients allocated to one of the MR-HIFU arms will not be able to undergo MR-HIFU and will therefore receive EBRT.

### Statistical analysis

All statistical analyses will be performed using IBM SPSS statistics, version 24 (IBM Corp. Armonk, NY, USA) and R version 4.0.4 (R foundation for statistical computing; https://R-project.org/). Data characterized by normal distribution will be expressed as means with standard deviations. Parameters not normally distributed will be expressed as medians with the interquartile ranges. Data will be analyzed according to the intention to treat and per-protocol principle. In case of post-treatment dropout (i.e., patients not surviving longer than a week, or patients unable to provide pain scores and analgesic use), a worst-case analysis will be performed, where dropped-out patients will be classified as non-responders.

The domain of the primary outcome is pain response measured by the Brief Pain Inventory (BPI [28, 29]) at baseline and 14 days following the start of treatment. Pain response is defined as a reduction of pain score ≥ 2 points without an increase of analgesic intake, or a reduction of analgesic intake of ≥ 25% without an increase of pain score at the treated site. The method of aggregation is the proportion of patients with a complete or partial pain response between baseline and 14 days following the start of treatment. Differences in pain response between arms will be compared by *χ*^2^-test. We will also conduct a time-to-event analysis for pain response. Here, the method of aggregation is the median time to pain response. Survival analysis will be performed by the Kaplan–Meier estimate and is calculated as the number of subjects surviving divided by the number of patients at risk (proportion). Survival proportions will be compared at 6 months. Toxicity will be presented as the proportion of CTCAE grade 3–4 toxicity in the 6 months following the start of treatment and differences will be tested with the *χ*^2^-test. QoL will be expressed as the mean (or median) score on a scale of 0–100 (EORTC [31–35]) and compared between the three groups at 3 and 6 months after treatment starts. A mean change of 10% of the scale breadth from the baseline will be considered a clinically relevant change in QoL. We will evaluate the pattern of QoL as a continuous outcome over time using mixed models. Differences with a *P*-value of < 0.05 will be considered statistically significant. No additional subgroup analyses are planned.

For the cost-effectiveness analysis, the primary outcome will be presented as cost per responder. Quality-adjusted life years (QALYs) will be derived from the EQ-5D-5L questionnaires to be applied in the cost-utility analysis. The costs of MR-HIFU and EBRT will be calculated using an actual cost accounting approach.

Adopting a payer perspective, we will calculate the incremental cost-effectiveness and cost-utility ratios at 6 months of follow-up. Deterministic and probabilistic (i.e., bootstrapping) sensitivity analysis will be conducted to test the robustness of the results. Because of the short timeframe, discounting will not be considered.

### Data—access, monitoring, and dissemination

The investigators, monitors, and employees of the Health and Youth Care Inspectorate (IGJ) may have access to the medical and research data of the patients. The responsibility of the IGJ is to supervise compliance of the trial with national and international legislation and regulations, mandated by Good Clinical Practice.

The responsibilities of the steering group are to independently assure scientific validity and monitor the progress of the study. The Medical Ethical Committee ensures the rights, safety, and well-being of FURTHER patients during the course of the trial and exists of patient representatives and a wide range of medical- and research-related disciplines. Study procedures and the quality of data are evaluated by independent monitors.

To ensure the confidentiality of patient data, local researchers of the participating centers can only access the data in the Castor EDC database of participants included at their own center. The Castor EDC database contains only pseudonymized data with the identification keys stored offline at the center where the patient is included.

A monitoring plan concerning a low-risk study has been implemented. Monitoring will be performed by the study sponsor (UMC Utrecht) and will include a site initiation visit, a yearly monitoring visit (after the inclusion of 2 patients), and a close-out visit for each participating center. Data collected from subjects treated in this trial will be used for conferences and publications unreservedly. Authorship on future publications will be considered for consortium members that actively participated in the realization of the study outcomes, and can be constrained to a fixed number per institute depending on journal guidelines. After study completion, data analysis, and reporting, data will be accessible for other research groups after irreversible deidentification.

No amendments to the trial have been applied. All future amendments will be submitted to the Medical Ethical Committee. Any changes in the protocol will be reported to all participating centers, and the funding partner through periodical consortium meetings.

### Patient involvement

During protocol writing, as for the interpretation of results, we collaborate with the patient sounding board group of the UMC Utrecht Cancer Center.

## Discussion

In this report, we present the rationale and design of the FURTHER trial. The aim of this international three-armed randomized controlled trial is to evaluate the effectiveness and cost-effectiveness of MR-HIFU—alone or in combination with EBRT—compared to EBRT as a palliative treatment option to relieve CIBP. Although the standard of care EBRT is a well-established treatment option, only approximately 55% of patients experience adequate pain relief after treatment [6]. MR-HIFU may improve pain palliation treatment for patients with painful bone metastasis, by providing fast and durable pain response in a higher percentage of patients [14]. Effectiveness and safety of MR-HIFU (alone or in combination with EBRT) as first-line treatment have never been compared to EBRT alone in an RCT.

MR-HIFU treatment provides a non-invasive, radiation-free treatment option for patients with CIBP caused by bone metastases. The treatment can be done in a single session and can be repeated multiple times if necessary. Preliminary clinical studies on the use of MR-HIFU for palliation of painful bone metastases demonstrated excellent response rates and safety [13–21]. Hurwitz et al. (2014) reported results of a multicenter randomized placebo-controlled trial to evaluate the safety and efficacy of MR-HIFU for treating bone metastases that were painful despite the previous radiotherapy, were unsuitable for radiotherapy, or who declined radiotherapy [14, 38]. Response to MR-HIFU was rapid, with about two-thirds of patients reporting pain response within a few days after treatment. Lee et al. (2017) performed a single-center matched-pair study which showed that MR-HIFU provides a similar overall treatment response rate but faster pain relief compared to EBRT and thus has the potential to serve as the first-line treatment for painful bone metastasis in selected patients [18]. Harding et al. (2018) evaluated QoL after MR-HIFU for painful bone metastasis and showed that there was a substantial positive effect on physical functioning and symptoms .[39]

MR-HIFU is considered a non-invasive low-risk intervention. There are however aspects of the treatment that make it a more complex treatment strategy for pain palliation. Currently, not all bone metastases are targetable with MR-HIFU (e.g., vertebrae). Close collaboration and good communication between the departments of radiation oncology and radiology are necessary for rapid referral and eligibility screening. In addition, patients will be under sedation during the MR-HIFU treatment. The use of sedation or general anesthesia for the MR-HIFU treatment has two major advantages. First, patients will lie completely still during the entire treatment, which decreases the risk of side effects and impaired treatment efficacy due to patient motion. Second, patients experience less discomfort and pain during MR-HIFU treatment. The risk of sedation is very low. However, to enable sedation, MR-HIFU treatment requires 1-day hospitalization, and some patients will have an extra hospital visit for the pre-procedural anesthesiologic screening. The planning of complex procedures in the palliative setting, where patients need to be treated as soon as possible, is challenging.

Since the planning of MR-HIFU is more complex and logistically more challenging than EBRT, we have incorporated patient-reported pain response at 14 days after inclusion as secondary endpoint. This way the FURTHER trial will provide insight into the real advantages and challenges of MR-HIFU from the patient’s perspective as well.

### Trial status

The FURTHER trial is registered under the Netherlands Trials Register number NL71303.041.19 and ClinicalTrials.gov registration number NCT04307914. Date of trial registration is 13–01-2020. This protocol is version 2 developed on 13 January 2020. The FURTHER trial has Ethical Approval in all participating centers and has started inclusion in multiple participating centers from 10–03-2020. Any protocol amendments will be tracked, dated, and logged in the trial registry. Recruitment is expected to be completed by 10–03-2023.

## Supplementary Information

**Additional file 1.** Informed consent form (Dutch version).

## Data Availability

All research data for this work are stored in an online repository and will be made available upon reasonable request to the corresponding author.

## Abbreviations

BPI: Brief Pain Inventory
CIBP: Cancer-induced bone pain
CT: Computed tomography
CTCAE: Common Terminology Criteria for Adverse Events
EBRT: External beam radiotherapy
ECOG: Eastern Cooperative Oncology Group
EDIZ: Ervaren Druk door Informele Zorg
EORTC C15-PAL: European Organisation for Research and Treatment of Cancer C15 Palliative questionnaire
EORTC QLQ BM22: European Organisation for Research and Treatment of Cancer Bone Metastases questionnaire
EQ-5D-5L: EuroQol 5-level evaluation questionnaire
FURTHER: Acronym: Focused Ultrasound and radioTHERapy for pain palliation in patients with painful bone metastasis
HADS: Hospital Anxiety and Depression Scale questionnaire
HIFU: High-intensity focused ultrasound
IMRT: Intensity-modulated radiation therapy
KPS: Karnofsky Performance Score
MR: Magnetic resonance
MR-HIFU: Magnetic resonance-guided high-intensity focused ultrasound
NRS: Numeric Rating Score
OMED: Oral morphine equivalence dose
PTV: Planning Target Volume
QoL: Quality of life
RT: Radiotherapy
SBRT: Stereotactic body radiation therapy
QALY: Quality-adjusted life years
SPSS: Statistical Package for the Social Sciences
VMAT: Volumetric modulated arc radiotherapy
WHO: World Health Organization

## References

[1] Mantyh PW (2014). Bone cancer pain: from mechanism to therapy. Curr Opin Support Palliat Care.

[2] Paice JA, Ferrell B (2011). The management of cancer pain. CA Cancer J Clin.

[3] Ripamonti C, Fulfaro F (2000). Malignant bone pain: pathophysiology and treatments. Curr Rev Pain.

[4] Ripamonti C, Fulfaro F. Pathogenesis and pharmacological treatment of bone pain in skeletal metastases. Q J Nucl Med. 2001;45(1):65–77. http://www.ncbi.nlm.nih.gov/pubmed/11456377. Accessed April 8, 2020. 11456377

[5] Fallon M, Giusti R, Aielli F, Hoskin P, Rolke R, Sharma M, Ripamonti CI, ESMO Guidelines Committee. Management of cancer pain in adult patients: ESMO clinical practice guidelines. Ann Oncol. 2018;29(Suppl 4):iv166–91. 10.1093/annonc/mdy152. 10.1093/annonc/mdy152 30052758

[6] Saito T, Toya R, Oya N (2019). Pain response rates after conventional radiation therapy for bone metastases in prospective nonrandomized studies: a systematic review. Pract Radiat Oncol.

[7] Chow E, Harris K, Fan G, Tsao M, Sze WM (2007). Palliative radiotherapy trials for bone metastases: a systematic review. J Clin Oncol.

[8] Van Der Linden YM, Lok JJ, Steenland E (2004). Single fraction radiotherapy is efficacious: a further analysis of the Dutch Bone Metastasis Study controlling for the influence of retreatment. Int J Radiat Oncol Biol Phys.

[9] van der Velden JM, van der Linden YM, Versteeg AL (2018). Evaluation of effectiveness of palliative radiotherapy for bone metastases: a prospective cohort study. J Radiat Oncol.

[10] Huisman M, Van Den Bosch MAAJ, Wijlemans JW, Van Vulpen M, Van Der Linden YM, Verkooijen HM (2012). Effectiveness of reirradiation for painful bone metastases: a systematic review and meta-analysis. Int J Radiat Oncol Biol Phys.

[11] Siedek F, Yeo SY, Heijman E (2019). Magnetic resonance-guided high-intensity focused ultrasound (MR-HIFU): technical background and overview of current clinical applications (Part 1). Fortschr Rontgenstr.

[12] Huisman M, ter Haar G, Napoli A (2015). International consensus on use of focused ultrasound for painful bone metastases: current status and future directions. Int J Hyperth.

[13] Scipione R, Anzidei M, Bazzocchi A, Gagliardo C, Catalano C, Napoli A (2018). HIFU for bone metastases and other musculoskeletal applications. Semin Intervent Radiol.

[14] Hurwitz MD, Ghanouni P, Kanaev SV (2014). Magnetic resonance-guided focused ultrasound for patients with painful bone metastases: Phase iii trial results Background. J Natl Cancer Inst.

[15] Bertrand A-S, Iannessi A, Natale R (2018). Focused ultrasound for the treatment of bone metastases: effectiveness and feasibility. J Ther Ultrasound.

[16] Liberman B, Gianfelice D, Inbar Y (2009). Pain palliation in patients with bone metastases using MR-guided focused ultrasound surgery: a multicenter study. Ann Surg Oncol.

[17] Napoli A, Anzidei M, Marincola BC (2013). MR imaging–guided focused ultrasound for treatment of bone metastasis. Radiographics.

[18] Lee HL, Kuo CC, Tsai JT, Chen CY, Wu MH, Chiou JF (2017). Magnetic resonance-guided focused ultrasound versus conventional radiation therapy for painful bone metastasis: a matched-pair study. J Bone Jt Surg - Am.

[19] Catane R, Beck A, Inbar Y (2006). MR-guided focused ultrasound surgery (MRgFUS) for the palliation of pain in patients with bone metastases–preliminary clinical experience. Ann Oncol.

[20] Huisman M, Lam MK, Bartels LW (2014). Feasibility of volumetric MRI-guided high intensity focused ultrasound (MR-HIFU) for painful bone metastases. J Ther Ultrasound.

[21] Gianfelice D, Gupta C, Kucharczyk W, Bret P, Havill D, Clemons M (2008). Palliative treatment of painful bone metastases with MR imaging–guided focused ultrasound. Radiology.

[22] Wattenberg MM, Fahim A, Ahmed MM, Hodge JW (2014). Unlocking the combination: potentiation of radiation-induced antitumor responses with immunotherapy. Radiat Res.

[23] Milani V, Noessner E, Ghose S (2002). Heat shock protein 70: role in antigen presentation and immune stimulation. Int J Hyperth.

[24] Hurwitz MD (2019). Hyperthermia and immunotherapy: clinical opportunities. Int J Hyperth.

[25] M.T.J Bartels M, Verpalen IM, Ferrer CJ, et al. Combining radiotherapy and focused ultrasound for pain palliation of cancer induced bone pain; a stage I/IIa study according to the IDEAL framework. Clin Transl Radiat Oncol. January 2021. 10.1016/j.ctro.2021.01.005 10.1016/j.ctro.2021.01.005 PMC7822778 33532631

[26] Chan A-W, Tetzlaff JM, Gøtzsche PC (2013). SPIRIT 2013 explanation and elaboration: guidance for protocols of clinical trials. BMJ..

[27] Castor EDC. Castor Electronic Data Capture. 2019. Available at: https://castoredc.com.

[28] Cleeland CS, Ryan KM (1994). Pain assessment: global use of the Brief Pain Inventory. Ann Acad Med Singapore.

[29] Wu JSY, Beaton D, Smith PM, Hagen NA (2010). Patterns of pain and interference in patients with painful bone metastases: a brief pain inventory validation study. J Pain Symptom Manage.

[30] Chow E, Hoskin P, Mitera G (2012). Update of the international consensus on palliative radiotherapy endpoints for future clinical trials in bone metastases. Int J Radiat Oncol Biol Phys.

[31] Chow E, Hird A, Velikova G (2009). The European Organisation for Research and Treatment of Cancer Quality of Life Questionnaire for patients with bone metastases: the EORTC QLQ-BM22. Eur J Cancer.

[32] Zeng L, Chow E, Bedard G, Zhang L, Fairchild A, Vassiliou V, et al. Quality of life after palliative radiation therapy for patients with painful bone metastases: results of an international study validating the EORTC QLQ-BM22. Int J Radiat Oncol Biol Phys. 2012;84(3):e337–42. 10.1016/j.ijrobp.2012.05.028. 10.1016/j.ijrobp.2012.05.028 22763028

[33] Groenvold M, Petersen MA, Aaronson NK (2006). The development of the EORTC QLQ-C15-PAL: a shortened questionnaire for cancer patients in palliative care. Eur J Cancer.

[34] Pilz MJ, Aaronson NK, Arraras JI, Caocci G, Efficace F, Groenvold M (2021). Evaluating the thresholds for clinical importance of the EORTC QLQ-C15-PAL in patients receiving palliative treatment. J Palliat Med.

[35] Echteld MA, Onwuteaka-Philipsen B, van der Wal G, Deliens L, Klein M (2006). EORTC QLQ-C15-PAL: The new standard in the assessment of health-related quality of life in advanced cancer?. Palliat Med.

[36] Sapareto SA, Dewey WC (1984). Thermal dose determination in cancer therapy. Int J Radiat Oncol Biol Phys.

[37] ter Haar G, Mueller P, Adam A (2012). Principles of high-intensity focused ultrasound. Interventional Oncology.

[38] Bitton RR, Rosenberg J, LeBlang S (2021). MRI-guided focused ultrasound of osseous metastases. Invest Radiol.

[39] Harding D, Giles SL, Brown MRD (2018). Evaluation of quality of life outcomes following palliative treatment of bone metastases with magnetic resonance-guided high intensity focused ultrasound: an international multicentre study. Clin Oncol.

