# Supplementary material for: Focused Ultrasound and RadioTHERapy for non-invasive palliative pain treatment in patients with bone metastasis: a study protocol for the three armed randomized controlled FURTHER trial
Source: Trials. 2022 Dec 29;23:1061. doi: 10.1186/s13063-022-06942-1 (PMC9798627; doi:10.1186/s13063-022-06942-1)
Supplement: Supplementary file 1 — Additional file 1. Informed consent form (Dutch version). [file 13063_2022_6942_MOESM1_ESM.pdf]

Exemplaar voor ziekenhuis *Subjectnummer:…………..*

Bijlage D: Toestemmingsformulier proefpersoon

De FURTHER studie - Behandeling van pijn bij botuitzaaiingen

Officiële naam: Gefocust ultrageluid en radiotherapie voor non-invasieve palliatieve pijn behandeling bij patiënten met botuitzaaiingen

- Ik heb de informatiebrief gelezen. Ook kon ik vragen stellen. Mijn vragen zijn voldoende beantwoord. Ik had genoeg tijd om te beslissen of ik meedoe.
- Ik weet dat meedoen vrijwillig is. Ook weet ik dat ik op ieder moment kan beslissen om toch niet mee te doen of te stoppen met het onderzoek. Daarvoor hoef ik geen reden te geven.
- Ik geef toestemming voor het informeren van mijn huisarts/specialist die mij behandelt dat ik meedoe aan dit onderzoek.
- Ik geef toestemming voor het opvragen van informatie bij mijn huisarts en/of specialist die mij behandelt.
- Ik geef toestemming voor het verzamelen en gebruiken van mijn gegevens voor de beantwoording van de onderzoeksvraag in dit onderzoek.
- Ik weet dat voor de controle van het onderzoek sommige mensen toegang tot al mijn gegevens kunnen krijgen. Die mensen staan vermeld in deze informatiebrief. Ik geef toestemming voor die inzage door deze personen.
- Ik geef toestemming voor het delen van gecodeerde gegevens met leden van het samenwerkingsverband.
- Ik geef toestemming voor het informeren van mijn huisarts en/of behandelend specialist van onverwachte bevindingen die van belang (kunnen) zijn voor mijn gezondheid.
- Ik geef toestemming om mijn gegevens op de onderzoekslocatie nog 15 jaar na dit onderzoek te bewaren..
- Ik geef ◻ **wel**
  ◻ **geen** toestemming om mijn onderzoeksgegevens gedurende de 15 jaar dat deze bewaard moeten blijven te gebruiken voor toekomstig onderzoek op het gebied van de behandeling van pijn bij botuitzaaiingen.
- Ik wil meedoen aan dit onderzoek.

Naam proefpersoon:

Handtekening: Datum: __ / __ / ____

Ik verklaar dat ik deze proefpersoon volledig heb geïnformeerd over het genoemde onderzoek.
Als er tijdens het onderzoek informatie bekend wordt die de toestemming van de proefpersoon zou kunnen beïnvloeden, dan breng ik hem/haar daarvan tijdig op de hoogte.

Naam onderzoeker (of diens vertegenwoordiger):

Handtekening: Datum: __ / __ / ____
